# Molecular mechanism of the intramembrane cleavage of the β-carboxyl terminal fragment of amyloid precursor protein by γ-secretase

**DOI:** 10.3389/fphys.2014.00463

**Published:** 2014-11-27

**Authors:** Maho Morishima-Kawashima

**Affiliations:** Laboratory of Neuroscience, Graduate School of Pharmaceutical Sciences, Hokkaido UniversitySapporo, Japan

**Keywords:** amyloid β–protein, γ-secretase, amyloid precursor protein, Alzheimer's disease, intramembrane proteolysis

## Abstract

Amyloid β-protein (Aβ) plays a central role in the pathogenesis of Alzheimer's disease, the most common age-associated neurodegenerative disorder. Aβ is generated through intramembrane proteolysis of the β-carboxyl terminal fragment (βCTF) of β-amyloid precursor protein (APP) by γ-secretase. The initial cleavage by γ-secretase occurs in the membrane/cytoplasm boundary of the βCTF, liberating the APP intracellular domain (AICD). The remaining βCTFs, which are truncated at the C-terminus (longer Aβs), are then cropped sequentially in a stepwise manner, predominantly at three residue intervals, to generate Aβ. There are two major Aβ product lines which generate Aβ40 and Aβ42 with concomitant release of three and two tripeptides, respectively. Additionally, many alternative cleavages occur, releasing peptides with three to six residues. These modulate the Aβ product lines and define the species and quantity of Aβ generated. Here, we review our current understanding of the intramembrane cleavage of the βCTF by γ-secretase, which may contribute to the future goal of developing an efficient therapeutic strategy for Alzheimer's disease.

## Introduction

Amyloid β-protein (Aβ) is a key molecule in the pathogenesis of Alzheimer's disease (AD), which is the most common dementia among elderly people and is characterized by memory loss and cognitive decline. The Aβ is a 37–43 amino acid hydrophobic protein that constitutes senile plaques, a neuropathological hallmark of AD (Reviewed in Selkoe, 2011). Among the various Aβ species with variable C-terminal lengths, Aβ42 is believed to be the most neurotoxic and aggregation-prone species (Iwatsubo et al., 1994; Kuperstein et al., 2010), and its production and deposition can be enhanced by familial AD (FAD)-associated mutations. Thus, the regulation of the Aβ produced is a current central issue in the therapeutics for AD; although it has not yet been successful (Extance, 2010).

Aβ is produced from β-amyloid precursor protein (APP) through successive cleavages mediated by two aspartyl membrane proteases, β- and γ-secretases. The ectodomain shedding of APP by β-secretase generates a 99 amino-acid β-carboxyl terminal fragment (βCTF), an immediate substrate for γ-secretase. The generated βCTF is then processed by γ-secretase within the transmembrane domain, releasing Aβ and the APP intracellular domain (AICD) (De Strooper et al., 2012). The latter cleavage has been enigmatic because the proteolysis occurs within the membrane, that is, in the hydrophobic environment of the lipid bilayer. γ-Secretase is a membrane-embedded atypical protease comprised of four integral membrane proteins: presenilin (PS) 1 or PS2, nicastrin, Aph-1, and Pen-2 (De Strooper et al., 2012). PS serves as the catalytic subunit (Wolfe et al., 1999). The three other members play a role in the stabilization and maturation of the complex. Nicastrin has also been implicated in the substrate binding (Shah et al., 2005). Besides the βCTF, γ-secretase cleaves many type I membrane proteins including the Notch receptor, which is responsible for cellular signaling during development and in adults (De Strooper and Annaert, 2010). Recent structural studies for γ-secretase revealed that two catalytic Asp residues on the transmembrane domains 6 and 7 of PS face the water-accessible hydrophilic environment and act to catalyze the substrate proteolysis (Sato et al., 2006; Tolia et al., 2006; Li et al., 2013). Water can gain access through the cavity surrounded by multiple transmembrane domains, as shown in other PS family proteases (Hu et al., 2011; Li et al., 2013) and also in γ-secretase (Lu et al., 2014) very recently. In contrast, the molecular mechanism underlying the intramembrane cleavage of a substrate by γ-secretase is less clear. A better understanding of how the βCTF is processed to Aβ through intramembrane proteolysis by γ-secretase may help establish an efficient disease modifying drug that specifically regulates Aβ42 production and/or does not have the adverse side effects derived from the suppression of other substrate cleavages (such as the Notch receptor) (Extance, 2010). The present review covers the recent progress in the understanding of the molecular mechanism of the intramembrane proteolysis of the βCTF by γ-secretase.

## γ-cleavage and ε-cleavage

### ε-cleavage, a novel form of cleavage

**Figure 1:**
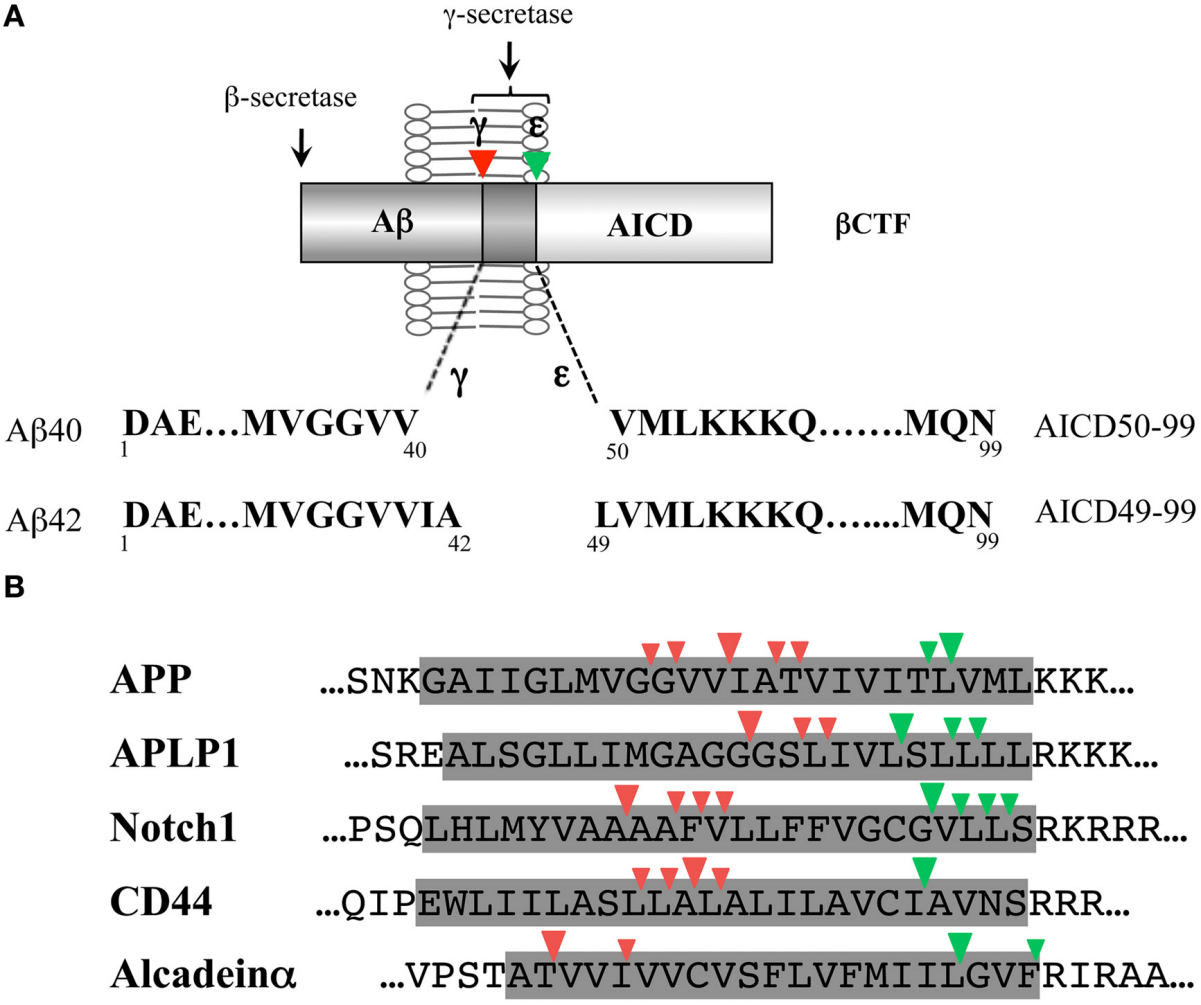
**γ-Cleavage and ε-cleavage by γ-secretase. (A)** Schematic illustration of γ- and ε-cleavages of the βCTF by γ-secretase. γ-Cleavage generates Aβ40 and Aβ42; while, ε-cleavage generates AICD50-99 and AICD49-99. There is a link between Aβ40 and AICD50-99 and between Aβ42 and AICD49-99. **(B)** Comparison of the γ-secretase-dependent intramembrane cleavage sites of various γ-secretase substrates. γ-Cleavages (site 4 cleavages) and ε-cleavages (site 3 cleavages) are shown by red and green arrowheads, respectively. The shaded area represents the predicted transmembrane domain. Either the human or rodent sequence is shown based on the identification studies. The three residue-spaced cleavages apply to the major γ- and ε-cleavage sites of APP, APLP1, Notch1, and CD44. In alcadeinα, γ- and ε-cleavages occur at three residue intervals, respectively.

Intramembrane cleavage of the βCTF by γ-secretase generates an Aβ of ~4 kDa and an AICD of ~6 kDa (Figure 1A). The AICD, a counterpart of the Aβ, is unstable in cells and has been postulated to start at either Ile41 or Thr43 (Aβ numbering). However, protein sequencing and mass spectrometric analysis of the AICD generated *in vitro* by cell-free or reconstituted Aβ generation systems revealed that the AICD starts at Val50 or Leu49 (Aβ numbering) (Gu et al., 2001; Sastre et al., 2001) and production of these AICDs was γ-secretase dependent. The novel cleavage to generate the AICD (referred to as ε-cleavage) (Weidemann et al., 2002) was located ~10 amino acids downstream of the Aβ generation sites (γ-cleavages), a few residues inside the membrane-cytoplasmic boundary, and is very similar to the site 3 cleavage of the Notch receptor (Schroeter et al., 1998). In the Notch signaling, γ-secretase-dependent Notch site 3 cleavage generates Notch intracellular domain (NICD) that mediates the signaling cascade in a variety of cell biological processes (De Strooper and Annaert, 2010), indicating the functional significance of this cleavage. Thus, γ-secretase cleaves the transmembrane domain of the βCTF in at least two sites: γ-cleavage generates Aβ while ε-cleavage generates the AICD. These dual cleavages are not inherent to the βCTF of the APP, but also occur in other γ-secretase substrates, such as APLP1/2 (Gu et al., 2001; Yanagida et al., 2009), Notch (Schroeter et al., 1998; Okochi et al., 2002; Tagami et al., 2008), CD44 (Okamoto et al., 2001; Lammich et al., 2002), and alcadeins α/β/γ (Hata et al., 2009; Piao et al., 2013) (Figure 1B).

### A potential link between γ- and ε-cleavages

The ε-cleavage is heterogeneous, similar to the γ-cleavage and the two molecular species of the Aβ and AICD that are generated appear to be linked (Figure 1A). In cells expressing wild-type APP and/or wild-type PS1/2, Aβ40 and AICD50-99 were predominant, and Aβ42 and AIDC49-99 were minor species. When various forms of FAD-mutant APP or FAD-mutant PS1/2 were expressed in cells, the proportion of Aβ42 vs. Aβ40 increased with a concomitant increase in the proportion of AICD49-99, although the relationship was not the same (Sato et al., 2003). A low concentration of the difluoro ketone peptidomimetic γ-secretase inhibitor DFK-167, (*N*-[(*S*)-2,2-difluoro-3-oxo-4-[(Boc-L-Val-L-Ile-)amino]pentanoyl]-L-Val-L-Ile-OMe), induced an increase in Aβ42, which also caused an increase in AICD49-99 (Sato et al., 2003). Thus, there is a link between Aβ40 and AICD50-99 and between Aβ42 and AICD49-99. A close relation between γ- and ε-cleavages was also suggested by the observation that APP FAD-mutations close to the ε-cleavage site (V717F, L723P) and the γ-cleavage site (T714I, V715A) influenced ε-cleavage as well as γ-cleavage, with remarkable increases in Aβ42 and AICD49-99 (Kakuda et al., 2006; Dimitrov et al., 2013).

### ε-cleavage precedes γ-cleavage

The potential link between Aβ42 and AICD49-99 raises a question: which cleavage, γ- or ε-, occurs first? It is likely that the ε-cleavage occurs first for the following reasons. First, the ε-cleavage site is located in close proximity to the cytoplasm, where water is available. In addition, the longest AICD detected so far in studies was AICD49-99. Thus, the ε-cleaved βCTFs of longer Aβs (Aβ49 and Aβ48) must then undergo γ-cleavage for Aβ generation. To test this proposal, Aβ49 and Aβ48 were overexpressed in cells and the molecular species of Aβ generated were investigated (Funamoto et al., 2004). The expression of Aβ49, a counterpart of AICD50-99, generated predominantly Aβ40; while, the expression of Aβ48, a counterpart of AICD49-99, preferentially produced Aβ42. These findings support the idea that ε-cleavage occurs first. Note that the expression of Aβ51, which is three residues longer than Aβ48, also produced predominantly Aβ42 (Funamoto et al., 2004). Thus, longer Aβs generated through ε-cleavage are processed to Aβ40/Aβ42 by γ-secretase. Moreover, the initial ε-cleavage sites determine the subsequent γ-cleavage sites and the type of Aβ species produced.

## Stepwise successive processing of longer Aβs by γ-secretase generates Aβ40 and Aβ42

### Longer Aβs are intermediate products present in cells

When ε-cleavage precedes γ-cleavage, longer Aβs should be produced. Thus, it is important to identify these intermediate molecules. The corresponding intermediates, which are Aβ species longer than Aβ1-42, are retained in the membrane in minimal amounts, if any. These Aβ species were immunoprecipitated with the AβN-terminus specific antibody from the membrane fraction and analyzed by Western blotting using a modified SDS/urea gel system that could distinguish Aβ37 through Aβ49, even when the Aβ species varied by only one residue (Qi-Takahara et al., 2005). Longer Aβs, including Aβ43, Aβ45, Aβ46, and Aβ48, were identified in the cells and in mouse brains. Their production was γ-secretase dependent. In our hands, Aβ49 was hardly detectable. In cells expressing wild-type APP and/or wild-type PS, the major intracellular Aβ species were Aβ40, Aβ43, and Aβ46, and the minor ones were Aβ42, Aβ45, and Aβ48. In cells expressing mutant APP or mutant PS, decreases in Aβ40 and increases in Aβ42 sometimes accompanied decreased levels of Aβ43 and Aβ46 and increased levels of Aβ45 and Aβ48; however, the coordination was not always obvious (Qi-Takahara et al., 2005).

**Figure 2:**
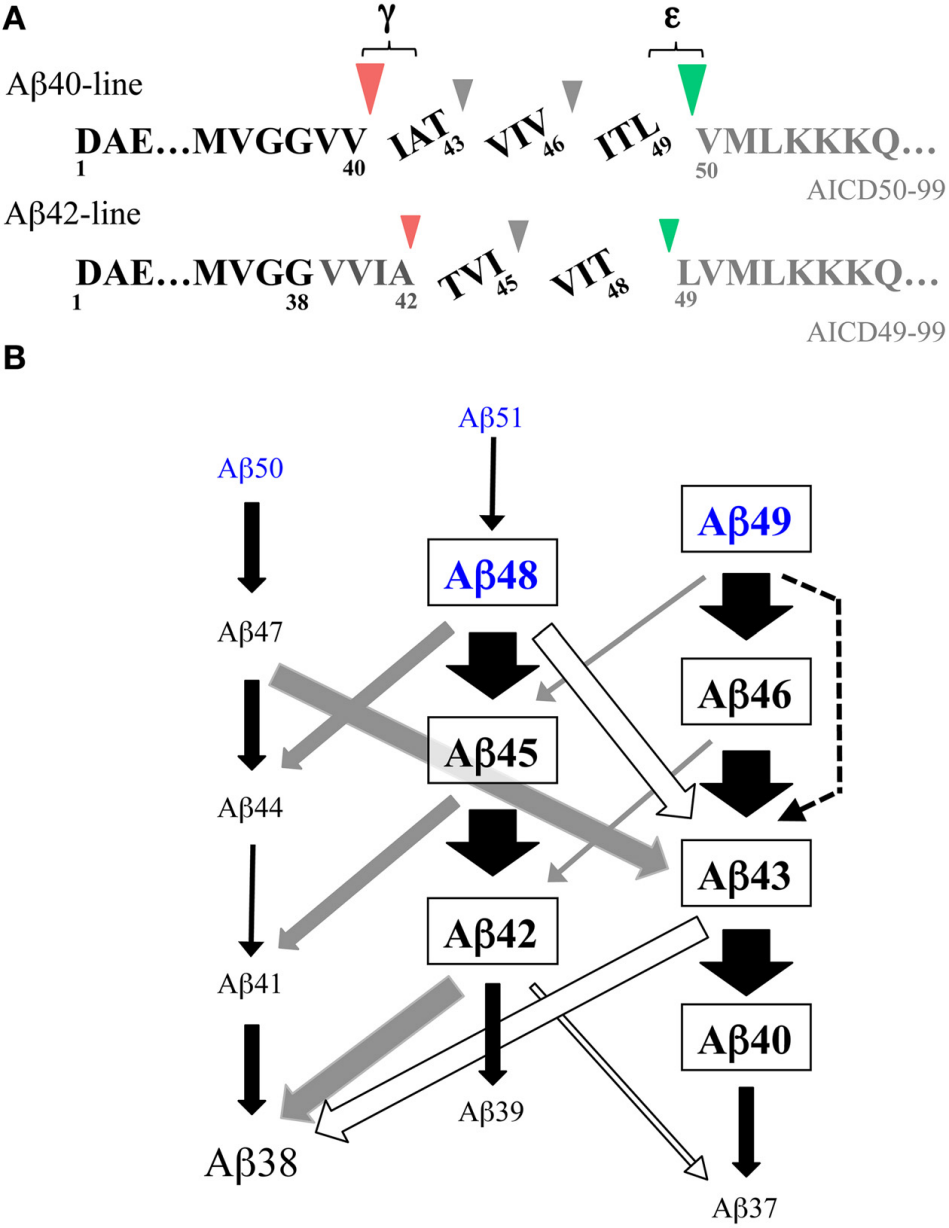
**Stepwise successive processing of the βCTF by γ-secretase generates Aβ. (A)** There are two major product lines generating Aβ40 and Aβ42, respectively. Aβ49, a major ε-cleaved product, is converted to Aβ40 through Aβ46 and Aβ43, which accompanies the release of the tripeptides ITL, VIV, and IAT. Aβ48, a minor ε-cleaved product, is converted to Aβ42 through Aβ45, releasing the tripeptides VIT and TVI. These pathways are estimated to represent ~75% of total Aβ production. Part of Aβ42 is further converted to Aβ38, releasing a tetrapeptide VVIA. Red and green arrowheads represent γ- and ε-cleavages, respectively. **(B)** Schematic illustration of the multiple interactive pathways for stepwise successive cleavages of the βCTF by membrane-integrated γ-secretase. The data on the peptides released by raft-associated γ-secretase (Matsumura et al., 2014) are summarized. Besides two major Aβ product lines shown in **(A)**, many alternative cleavages occur, releasing peptides with three to six residues. These minor routes link the two major pathways in an interactive manner and modulate Aβ production. It is likely that ε–cleavage generates trace amounts of Aβ51 and Aβ50 in addition to Aβ49 and Aβ48 (blue letters) (see Sato et al., 2003; Olsson et al., 2014). Black, gray, and white arrows represent the release of tri-, tetra-, and penta-peptides, respectively. A dotted arrow represents the release of a hexapeptide. The relative thicknesses of the arrow represent the amount of the released peptides. Note that most Aβ38 is generated either from Aβ42 or Aβ43 by the release of a tetrapeptide or pentapeptide.

Thus, γ-secretase cleaves the transmembrane domain of the βCTF at multiple sites (Figure 2A). The cleavage site between the γ- and ε-cleavage sites is called the ζ-site (Zhao et al., 2004). These cleavage sites appear to be divided into two groups, the sites relevant to Aβ40 production (Aβ43, Aβ46, Aβ49) and those relevant to Aβ42 production (Aβ45, Aβ48). The cleavage at three residue intervals is a prominent property in each group. This notion is supported also by the observation that the V721K APP mutation led to increased AICD47-99 (a counterpart of Aβ46) and a concomitant increase in Aβ40.

### Stepwise successive processing by γ-secretase generates Aβ

Interestingly, dose-dependent treatment with DAPT (*N*-[*N*-(3,5-difluorophenacetyl)-L-alanyl]-*S*-phenylglycine *t*-butyl ester), a potent dipeptide γ-secretase inhibitor, caused differential accumulations of longer Aβs in the cells that inducibly expressed the βCTF (Qi-Takahara et al., 2005). A decrease in Aβ40 levels accompanied a transient increase in Aβ43, which, in turn, brought about a transient increase in Aβ46. One plausible explanation for this observation would be the precursor-product relationship. Suppression of Aβ40 results in accumulation of its precursor molecule, Aβ43, and subsequently, suppression of Aβ43 induces accumulation of its precursor Aβ46. Thus, it is reasonable to speculate that Aβ40 is produced successively from Aβ46 through Aβ43. In contrast to the wild-type cells, dose-dependent treatment with DAPT did not induce dramatically different intracellular accumulations of Aβ in a consistent manner in mutant PS cells (Yagishita et al., 2006). PS2 cells with the N141I mutation exhibited a remarkable decrease in Aβ42 and a concomitant increase in Aβ45, while M233T mutant PS1 cells showed a decrease in Aβ42, which accompanied a slight transient increase in Aβ48.

These results led us to propose the stepwise successive processing model by γ-secretase for Aβ generation (Qi-Takahara et al., 2005) (Figure 2A). In this model, γ-secretase cleaves the βCTF first at the ε-cleavage site close to the membrane-cytoplasm boundary and the truncated βCTF (longer Aβs) generated is processed from the C-terminus at every third residue. According to the model, Aβ49, a major ε-cleaved product, is converted to Aβ40 through Aβ46 and Aβ43, releasing the tripeptides ITL, VIV, and IAT. The other minor ε-cleaved product, Aβ48, is converted to Aβ42 through Aβ45, releasing the tripeptides VIT and TVI. Consistent with this model, treatment of cells with DAPT caused accumulation of Aβ46 in lipid rafts, which was processed to Aβ40 and Aβ43, but not Aβ42, in a γ-secretase-dependent manner through *in vitro* incubation of the isolated rafts (Yagishita et al., 2008).

### Tripeptides are released concomitantly with Aβ generation

The identification of the tripeptides released by γ-secretase during Aβ generation provides convincing evidence for this cleavage model. These tripeptides were directly identified and quantified in the reaction mixture of a CHAPSO-solubilized reconstituted γ-secretase system using liquid chromatography with tandem mass spectrometry (LC-MS/MS) (Takami et al., 2009); in this system, the βCTF purified from Sf9 cells was used as a substrate. The predicted five tripeptides were all identified by LC-MS/MS. Three tripeptides in the putative Aβ40-product line (IAT, VIV, and ITL) and two tripeptides in the putative Aβ42-product line (TVI and VIT) were released concomitantly with Aβ generation. Additionally, a released tetrapeptide, VVIA, was identified, although in relatively low amounts (Figure 2A). This finding indicated that a part of Aβ42 is converted to Aβ38 by releasing VVIA. The release of those peptides was suppressed by γ-secretase inhibitors, indicating that their generation was γ-secretase-dependent. Similar tri- and tetrapeptides were released using synthetic Aβ peptides as substrates (Okochi et al., 2013). The quantification of the released peptides further validated the accuracy of the model (Takami et al., 2009). The relative relationships of the peptides were: ITL > VIV > IAT and VIT > TVI >> VVIA, which fitted the model. The Aβ levels estimated by the tripeptide amounts, according to the model, corresponded well with the levels determined by Western blotting. Thus, the proposed stepwise processing model is reasonable and there are two product lines: Aβ49 -> Aβ46 -> Aβ43 -> Aβ40 and Aβ48 -> Aβ45 -> Aβ42 (-> Aβ38) (Figure 2A).

### Multiple interactive pathways for stepwise successive processing generate Aβ

Lipid rafts are detergent-resistant membrane microdomains enriched in cholesterol and sphingolipids and play a significant role in Aβ generation in cells (Vetrivel and Thinakaran, 2010). These rafts exclusively contain all four components required for the active γ-secretase complex: the PS N-terminal fragment/C-terminal fragment, mature nicastrin, Aph-1, and Pen-2 (Wada et al., 2003; Vetrivel et al., 2004), indicating that active γ-secretase is present in lipid rafts (Hur et al., 2008). In addition, the lipid composition in the membrane of the rafts is favorable for γ-secretase activities: both cholesterol and sphingolipids have been shown to enhance its activities (Osenkowski et al., 2008). Thus, these lipid rafts can provide the proper lipid environment for Aβ generation, as seen in the *in vitro* Aβ generation systems, which exhibited higher γ-secretase activity in lipid rafts (Wada et al., 2003; Hur et al., 2008).

The membrane integrity of γ-secretase was not considered in the studies discussed above. Therefore, we assessed whether membrane-integrated γ-secretase followed the cleavage model using γ-secretase associated with lipid rafts. The reaction mixture of the *in vitro* reconstituted γ-secretase system with lipid raft-associated γ-secretase was subjected to LC-MS/MS analyses to identify the small peptides released from the transmembrane domain of the βCTF during Aβ generation (Matsumura et al., 2014). Similar to the CHAPSO-solubilized system, the predicted 5 tripeptides, IAT, VIV, ITL, TVI, and VIT, and the tetrapeptide, VVIA, were released in a γ-secretase-dependent manner with Aβ generation. The same quantitative relationships, ITL>VIV>IAT and VIT>TVI>>VVIA, were also present. Thus, raft-associated γ-secretase cleaves the transmembrane domain of the βCTF in a stepwise successive manner at every third or fourth residue to generate Aβ40 and Aβ42 (Aβ38). However, Aβ generation by raft-associated γ-secretase accompanied the release of novel penta- and hexapeptides, as well as tri- and tetrapeptides. Although they were in low amounts and the original two pathways that generated Aβ40 and Aβ42 amounted to ~75% of total Aβ production (Matsumura et al., 2014), the clipping of the novel peptides, in particular pentapeptides, links the above two major pathways at several points and allows for an alternative route for successive cleavages (Figure 2B). The presence of multiple interactive pathways for the stepwise cleavages of the βCTF could modulate the nature of the species and the quantity of Aβ generated. In fact, these interactive pathways could provide a better explanation for several previous studies apparently inconsistent with the model that affords two Aβ product lines (see Matsumura et al., 2014). Similar three- to six-residue peptides were also released and identified in the cell-free Aβ generation system with an endogenous substrate, where no detergent was used (Olsson et al., 2014). In this study, small amounts of Aβ40 and Aβ42 continued to be processed in a stepwise manner, being further converted into smaller Aβs such as Aβ37, Aβ36, and Aβ34 (Olsson et al., 2014). It is worth noting that Aβ38 and Aβ43 may be generated via three routes, releasing a tri-, tetra-, and penta-peptide, respectively, (Figure 2B). γ-Secretase modulators (GSMs), such as GSM-1 ((2*S*,4*R*)-1-[(*R*)-1-(4-chlorophenyl-4-methylpentyl)-2-(4-trifluoromethylphenyl)piperidin-4-yl]acetic acid), can selectively lower Aβ42 levels and are a prospective therapeutic tool for AD. These modulating compounds enhanced all three routes that generate Aβ38 and the conversion of Aβ40 to Aβ37. Significant decreases in conventional Aβs (Aβ42, Aβ43, and Aβ40) occurred as well as increases in the levels of shorter Aβs (Aβ38 and Aβ37) (Takami et al., 2009; Okochi et al., 2013; Matsumura et al., 2014; Olsson et al., 2014). Thus, the influence of GSMs is not limited to a single pathway (the conversion of Aβ42 to Aβ38), but advances the stepwise cleavage by γ-secretase one step further, generating shorter Aβ species.

It is possible that in the proper lipid environment, γ-secretase favors certain cleavage sites over others, resulting in differences in cleavage products when the protease is CHAPSO-solubilized vs. membrane-integrated. Cholesterol may modulate the cleavage specificity of γ-secretase (Osenkowski et al., 2008). However, a pentapeptide VVIAT was released by CHAPSO-solubilized γ-secretase with large amounts of synthetic Aβ43 as a substrate (Okochi et al., 2013). Thus, it is more likely that the variable cleavages that occur as a consequence of the surrounding conditions are an inherent property of γ-secretase. When γ-secretase had higher activity in the membrane environment, a number of co-released minor peptides would be easily identified.

Most FAD mutations on PS impair the γ-secretase activities. Some mutations on PS1 reduced the cropping activity of γ-secretase (Okochi et al., 2013; Fernandez et al., 2014) and led to accumulation of longer Aβs such as Aβ43, Aβ45, and Aβ46 (Shimojo et al., 2008; Quintero-Monzon et al., 2011). While Aβ43 is another neurotoxic Aβ species (Saito et al., 2011), the toxicity and aggregation properties of Aβ45 and Aβ46 are not yet understood. However, it is possible that the accumulation of longer Aβ induces further impairment of the γ-secretase function and accelerates the disease progression. The observation by Yagishita et al. (2008) that Aβ46 accumulated in the presence of DAPT was converted to Aβ40 and Aβ43 even in the presence of L-685,458 ([1*S*-benzyl-4*R*-(1*S*-carbamoyl-2-phenylethylcarbamoyl-1*S*-3-methylbutylcarbamoyl)-2*R*-hydroxy-5-phenylpentyl] carbamic acid *tert*-butyl ester), a transition state analog γ-secretase inhibitor, indicated that Aβ46 generated as an intermediate remains bound to the catalytic site of γ-secretase. The altered binding kinetics of Aβ46 may result in disturbed turnover of γ-secretase.

Proteolytic cleavage of the α-helix generally requires local unwinding to expose a scissile peptide bond to the catalytic site of the protease. The initial endopeptidase-like cleavage of the βCTF by γ-secretase may be facilitated by the flexible loose structure around the ε-cleavage site, which has been revealed by NMR (Sato et al., 2009; Lu and Tycko, 2011). Since ε-cleaved long Aβ (Aβ49 or Aβ48) is hardly detectable in any system (Qi-Takahara et al., 2005; Kakuda et al., 2006), it is likely that cropping proceeds swiftly in the same cellular compartment (Qi-Takahara et al., 2005), once ε-cleavage is initiated. The cleavage sites aligned on the surface of the α-helix of the βCTF transmembrane domain (~3.6 residues for one turn of α-helix) may encourage the recognition and/or proteolysis by γ-secretase (Qi-Takahara et al., 2005). The observations of the major three-residue spaced stepwise processing and the differential sensitivity to DAPT observed among cleavage sites are consistent with this assumption (Qi-Takahara et al., 2005). On the other hand, the release of tetra- and pentapeptides, in addition to tripeptides, may also support the theory that the fraying helix terminus generated by the cleavage promotes the next cleavage (Sato et al., 2009; Pester et al., 2013). Future structural studies may uncover hidden enzymatic properties of γ-secretase, as the recent identification of a structure homologous to carboxypeptidase within the structure of nicastrin (Lu et al., 2014). The termination of the stepwise processing should release Aβ. Glycine residues in the transmembrane domain may determine the termination point (Munter et al., 2007; Pester et al., 2013; Lemmin et al., 2014). Alternatively, Aβ may be released due to decreased hydrophobicity of the shortened Aβ stub. The absence of glycine residues in the Notch and CD44 transmembrane domain (Figure 1B) supports the latter possibility. Either way, the primary cleavage site (γ-, ε-, or ζ-) appears to be critical in determining the final Aβ species produced. The properties of amino acids lining the cleavage sites are also important in determining the cleaved residues, because substitutions of those amino acids generated alternative cleavage sites (Lichtenthaler et al., 1999; Sato et al., 2005).

## Concluding remarks

The successive tripeptide-cropping pathway is the basal framework for the βCTF cleavage by the membrane-integrated γ-secretase, but many alternative cleavages occur to release three- to six-residue peptides. There is crosstalk between the pathways involved in stepwise successive processing for Aβ generation by γ-secretase. The stepwise sequential processing mechanism may be a general property of intramembrane proteolysis by the γ-secretase family of proteases (see Figure 1B). Several residue-spaced cleavages have also been identified in PS (Fukumori et al., 2010) and in tumor necrosis factor-α (a substrate of signal peptide peptidase-like protein) (Fluhrer et al., 2006). Cleavage at three residue intervals appears to be favorable at least for γ-secretase, but it is not required. The stepwise successive processing by γ-secretase may be at work to metabolize various membrane-spanning proteins in the membrane as with the proteasome in the cytoplasm (Kopan and Ilagan, 2004), since small peptides are promptly released from the membrane. On the other hand, γ-secretase-mediated endoproteolysis plays a critical role in cellular signaling: shedding-primed ε-like cleavage modulates cellular signaling pathways through the released C-terminal intracellular domain (ICD), as typically observed in the Notch receptor (De Strooper and Annaert, 2010). Thus, γ-secretase may have two distinct physiological functions coupled with the proteolysis. In addition, both functions may be coordinated.

### Conflict of interest statement

The author declares that the research was conducted in the absence of any commercial or financial relationships that could be construed as a potential conflict of interest.
